# Adrenal Cysts: To Operate or Not to Operate?

**DOI:** 10.3390/jcm13030846

**Published:** 2024-02-01

**Authors:** Ivana Bozic Antic, Igor Djurisic, Srdjan Nikolic

**Affiliations:** 1 Department of Endocrinology, Euromedik General Hospital, 11000 Belgrade, Serbia; 2 Faculty of Dentistry Pancevo, University Business Academy, 21000 Novi Sad, Serbia; 3 Institute for Oncology and Radiology of Serbia, 11000 Belgrade, Serbia; 4 Faculty of Medicine, University of Belgrade, 11000 Belgrade, Serbia

**Keywords:** adrenal cyst, endothelial, adrenal gland surgery, SARS-CoV-2, COVID-19

## Abstract

Adrenal cysts are uncommon and usually asymptomatic, and therefore are usually incidentally discovered adrenal lesions. They have a broad pathohistological spectrum that includes pseudocysts and endothelial (vascular), parasitic, and epithelial (mesothelial) cysts. Although most adrenal cysts are benign and hormonally non-functional lesions, some can have ambiguous imaging appearances and mimic malignant adrenal neoplasms. On the other hand, the actual malignant neoplasms could undergo cystic transformation. Additionally, immune cell infiltrations, thrombosis, or haemorrhage seen in sepsis can frequently cause adrenal cyst development, raising a question about the possible connection between severe acute respiratory syndrome coronavirus type 2 (SARS-CoV-2) and adrenal cystic lesions. Due to the disease’s rarity, the likelihood of malignancy, and the lack of specific guidelines, the management of adrenal cysts is always challenging especially in a young person. This review discusses the important diagnostic and the current treatment possibilities for adrenal cystic lesions. Aiming to emphasize clinical dilemmas and help clinicians navigate the challenges when encountering a patient with an adrenal cyst in everyday practice, we based our review on a practical question–answer framework centred around the case of a young woman with an incidentally discovered large adrenal cyst.

## 1. Introduction

Adrenal cysts are a rare phenomenon in the pathology of the adrenal gland, with a reported overall incidence in the range of 0.064% to 0.18% and accounting for roughly 4% of all adrenal masses [1,2,3]. Most of them are benign, hormonally non-functional lesions that are symptomatic in only 10% of cases, have a slight female predominance, and are bilateral in approximately 3–8% of cases [4,5,6]. The size of an adrenal cyst can range from 0.5 cm to 20 cm (a median size of 4.8 cm) [6]. In the past, large adrenal cysts were usually discovered due to their mass effect symptoms (pain and abdominal discomfort). However, the increasing use of diagnostic imaging in the past few decades led to a higher incidence of adrenal incidentalomas of which 1–2% were adrenal cysts [6,7,8]. Also, adrenal cyst presentation has changed—they now tend to be diagnosed at their earlier stage of development, being smaller and asymptomatic [9]. Although most adrenal cysts are benign and hormonally non-functional lesions, some can have ambiguous imaging appearance and mimic malignant adrenal neoplasms [1]. On the other hand, the actual malignant neoplasms could undergo cystic transformation [1]. Any adrenal tumour larger than 5 cm is referred to as a large adrenal tumour (LAT), regardless of whether it has undergone a cystic transformation. LATs are rather rare, with a frequency ranging from 8.6 to 38.6% [7,10,11]. The likelihood of malignancy rises with the increasing volume of adrenal mass [11,12]. In the large series of surgically treated LATs, benign tumours were present in 62–68% cases, while malignant tumours accounted for 28–37% of cases [11,12]. Therefore, rigorous preoperative evaluation is crucial for making the right treatment decision, especially as there is no specific guideline for managing adrenal cysts yet.

## 2. Materials and Methods

We reviewed all case reports, review articles, and clinical studies regarding adrenal cysts published in PubMed in the last 10 years. In our research, we used the keyword “adrenal cyst”. The paucity of review papers and the existence of a few studies involving a larger number of patients with adrenal cysts were not unexpected. Case reports and a few case studies still comprise the bulk of the published material. Aiming to emphasize clinical dilemmas and help clinicians navigate the challenges when encountering a patient with an adrenal cyst in everyday practice, we based our review on a practical question–answer framework centred around the case of a young woman with an incidentally discovered large adrenal cyst.

## 3. Case Description

**Figure 1:**
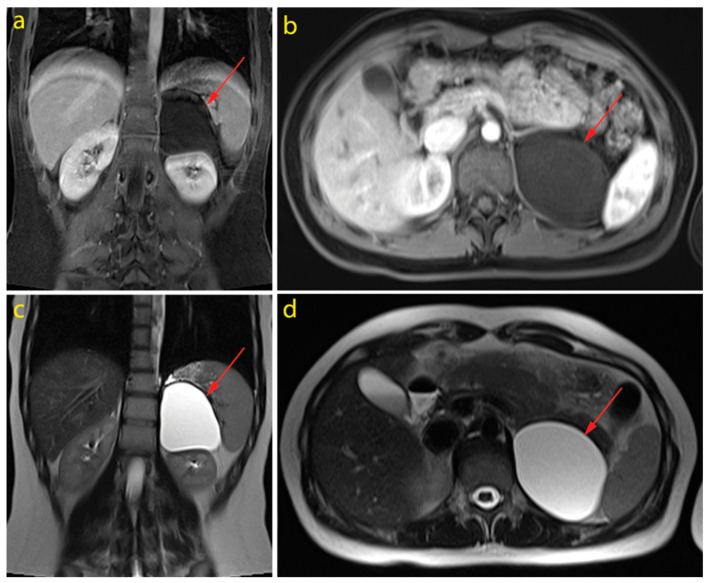
Magnetic resonance imaging (MRI) of the cystic tumour in the left adrenal lodge: T1-weighted sequence (**a**,**b**) and T2-weighted sequence (**c**,**d**), on the coronary (**a**,**c**) and the axial (**b**,**d**) plane.

An 18-year-old clinically asymptomatic woman underwent a self-initiated medical checkup that included her first-ever abdominal ultrasound examination one month after being diagnosed with mild coronavirus disease 2019 (COVID-19). She was treated only with symptomatic therapy. An abdominal ultrasound examination revealed a large retroperitoneal cystic tumour mass above the upper pole of the left kidney. Magnetic resonance imaging (MRI) showed an oval cyst with a thin wall in the lodge of the left adrenal gland, measuring 60 × 78 mm in the axial plane, and 80 mm in the craniocaudal plane. The cyst content had a lower T1-weighted and higher T2-weighted signal and no diffusion restriction. There was a discrete rim amplification of signal intensity after the contrast application. The tumour mass pushed the pancreas and the left kidney and was in close contact with the spleen and both left adrenal gland cruses (Figure 1).

There were no clinical abnormalities during the physical examination, her body mass index was 20 kg/m^2^, and her blood pressure was 120/70 mmHg. Her nasopharyngeal swab was negative for severe acute respiratory syndrome coronavirus 2 (SARS-CoV-2). ECG and chest X-ray were normal. The tumour mass was not palpable. All hormonal analyses were unremarkable (Table 1). 

The patient’s parents accepted the recommendation of surgical therapy made by the tumour board, consisting of an endocrinologist, a surgeon, and an oncologist. Surgery was performed laparoscopically with the transperitoneal approach. The cyst was extirpated in total, and the left adrenal gland remained intact. The analysis of cyst content revealed erythrocytes, granulocytes, protein, and normal endothelial cells without atypia. The outer side of the cyst capsule was smooth, glowing, and whitish while the inner side was uneven, yellowish, and elastic. Immunohistochemistry revealed that the cyst capsule contains a monolayer of normal endothelial cells, which stained positive for CD31, CD34, and factor VIII and negative for WT1, CK5/6, calretinin, and EMA, while there was focal positivity for AE1/AE3. The final diagnosis was an endothelial cyst of the adrenal gland. The patient fully recovered, and there was no need for hormone replacement therapy.

## 4. Discussion

### 4.1. Question 1: What Is the Epidemiology of Adrenal Cysts?

Adrenal cysts are rare and frequently asymptomatic, and therefore are usually incidentally discovered lesions. According to studies that involved a large number of patients, adrenal cysts comprise 1–2% of all adrenal incidentalomas and have slight female predominance [6,7,8,9]. Benign adrenal cysts are usually discovered incidentally (in 88% of cases), but a significant number (up to 40% of cases) of malignant adrenal neoplasms with cystic transformation are also incidentalomas [6,9,13]. Adrenal cysts used to be the most prevalent in the fourth to seventh decade of life [6,9,14], but the more frequent use of imaging diagnostics has led to the shift in the age range to younger patients (fourth to fifth decade of life) [3,6,14,15]. Our patient was very young (18 years old) at the time of diagnosis of the large adrenal cyst (8 cm), which makes her one of the youngest cases with this kind of pathology. Unusual pathology, especially in a young patient, frequently raises the suspicion of possible malignancy. Furthermore, it should be the first concern of a clinician who encounters a patient with an adrenal cyst. 

### 4.2. Question 2: Does the Adrenal Cyst Size Matter?

According to the largest series of benign adrenal cysts published in 2022, the median cyst size range is 0.5–20 cm [6]. Younger patients (median age: 37) usually have larger adrenal cysts (>5 cm) in comparison to older patients (median age: 51) who have smaller (<5 cm) adrenal cysts [6]. Malignant adrenal cystic neoplasms are usually larger, with a median size range of 3.6–28 cm [9,13,14,16]. As the possibility of malignancy increases with tumour size [11,12], some societies have been suggesting that an additional criterion for the surgical treatment of adrenal incidental tumours should be an adrenal tumour size of more than 5 cm [17]. However, the new guideline for the management of adrenal incidentalomas provides preference to the imaging characteristics of the adrenal tumour over its size [18]. Concerning the previously cited data, we could anticipate that a young patient such as ours would have a large benign adrenal cyst, yet the size of the cyst did not allay our fears of it being potentially cancerous.

### 4.3. Question 3: To What Extent Can We Rely on Imaging?

Benign adrenal cysts usually appear as oval, well-demarcated masses with thin walls. They are homogenous, anechoic, or hypoechoic on ultrasound, while on MRI, they have low signalling on T1-weighted and high signalling on T2-weighted sequences [6]. On computed tomography (CT), they usually have low attenuation (0–20 Hounsfield Units), which classifies them as benign or intermediate adrenal masses [6,18]. Except for the cyst wall, which sometimes can captivate contrast, other parts of the cyst usually do not show contrast enhancement [6,19]. However, even benign adrenal cysts could have a broad range of unenhanced CT attenuation and heterogenous appearance due to haemorrhage, as well as irregular shapes when compressing other adjacent anatomic structures, which sometimes makes it a real challenge to differentiate them from malignant neoplasms. The adrenal cyst in our case had benign MRI characteristics; however, clinicians should not rely only upon the radiological appearance of adrenal cysts when deciding if the mass is benign or not. Rather, the lack of contrast enhancement and the cyst’s unaltered size and form on a subsequent follow-up imaging after six months often indicate a benign adrenal cyst’s most likely diagnosis [9,18].

### 4.4. Question 4: Which Adrenal Cyst Subtypes Exist?

The broad pathohistological spectrum of adrenal cysts includes pseudocysts and endothelial (vascular), parasitic, and epithelial (mesothelial) cysts [1]. In surgical series, pseudocysts are the most frequently encountered (up to 80%), while endothelial cysts are the most frequent pathology in the autopsy series (up to 45%) [1,2,16,20,21,22]. The key feature of the pathohistological classification of adrenal cysts is the cyst wall structure. In contrast to other adrenal cyst subtypes, only pseudocysts do not contain cell lining in the cyst wall [1]. 

Pseudocysts are complex cysts that can develop in previously healthy adrenal glands (due to haemorrhage induced by trauma, toxins, or infection) or in adrenal neoplasms (due to degenerative necrosis or haemorrhage) [9,16]. Their imaging appearance is usually complex and variable and can mimic abscesses, carcinomas, and metastases [23]. Histologically, pseudocysts are surrounded by a thick, fibrous capsule that has no internal cell lining, while the inner part of the cyst contains blood and fibrin [1]. 

Endothelial cysts are presumed to be the second-most common adrenal cystic lesions, with a prevalence of 45% in the autopsy series [21] and 2–24% in the surgical series [1,24]. They are simple cysts, arising from pre-existing vascular or lymphatic malformations, and are rarely associated with malignant neoplasms [1]. According to the literature and in contrast to our case, endothelial cysts are usually up to 2 cm in diameter [25]. On MRI, these thin wall cysts have low T1 and high T2 intensity, as was with our case, and rim contrast enhancement is usually due to the compression of peripheral adrenal tissue [26,27]. Their capsule contains a monolayer of endothelial cells [1]. 

Epithelial adrenal cysts are most rarely encountered, with an estimated incidence of 6–9% [22,28]. In contrast to both pseudocysts and endothelial cysts, which are vascular-origin cysts, epithelial cysts are non-vascular, and their pathogenesis is yet unknown [1,25]. Their capsule is usually thin and lined by flat to cuboidal cells [1]. 

Parasitic adrenal cysts are usually of echinococcal origin, occurring as a part of the disseminated hydatid disease, while the isolated adrenal hydatid cysts are extremely rare [29]. Positive hydatid serology and eosinophilia are not always present, so the suspicion of parasitic adrenal cysts is often clinical, while a frequently specific appearance on imaging (“cysts-within-a-cyst” and peripheral calcifications) could help [29,30]. Histologically, the parasitic cyst capsule contains eosinophilic granulocytes, while the insides contain parasites, which can be sometimes calcified [29].

Immunohistochemical profiling is usually not needed for pseudocysts and parasitic cysts but often can help in the differentiation of epithelial (positive stain for calretinin and WT1) and endothelial cysts (positive stain for CD31, D2-40, and ERG) [1].

### 4.5. Question 5: Can Adrenal Cysts Be Hormonally Active?

In terms of hormonal secretion, true adrenal cysts are non-functional tumours [3,6,9]. However, primary and secondary adrenal tumours can undergo a cystic transformation due to intratumour haemorrhage or necrosis. Accordingly, the possible hormonal functionality and malignancy of adrenal cystic lesions should be always kept in mind. Cysts with a median size of greater than 5 cm are considered to be at a higher risk of malignancy and are usually presented as pseudocysts [5,9]. Despite our patient’s cyst being large (8 cm), the MRI showed it to be a straightforward simple cyst without any concerning features. Nevertheless, every patient diagnosed with an adrenal mass, including a cystic one, requires a hormonal evaluation. 

Eliminating the possibility of pheochromocytoma is the first and most crucial step in the evaluation of any adrenal tumour, including cystic ones. There are several reasons why this is so important. As most of these tumours hypersecrete catecholamines, cardiovascular morbidity and mortality are high in patients with untreated pheochromocytomas [31,32]. Furthermore, there is a significant intraoperative risk for cardiovascular events if the patient with an unrecognized pheochromocytoma is not appropriately prepared for the surgery [31,32,33]. The potential to grow and induce mass effect symptoms, the considerable prevalence of malignancy (15–25% of cases), and the possibility of hereditary disease (up to 30% of cases) should also urge a clinician to thoroughly investigate the likelihood of pheochromocytoma when encountering a patient with an adrenal tumour [31,34].

According to the current guidelines, the initial testing for pheochromocytoma includes the biochemical analyses of catecholamine metabolites: urinary fractionated metanephrines or plasma-free metanephrines [31,35]. These analyses should be performed in every patient with an adrenal tumour regardless of the clinical symptoms of catecholamine excess. However, the normal biochemical tests do not rule out the possibility of pheochromocytomas as a considerable proportion of patients (up to 30%) could have not only a clinically silent but also a non-secretory pheochromocytoma [36]. This is frequently the case with cystic pheochromocytomas as only one-third of patients with cystic pheochromocytomas have the symptoms of catecholamine excess [13,37,38]. Cystic pheochromocytomas are rarely encountered tumours (approximately 3% of all pheochromocytomas and 0.27% of all adrenal tumours), diagnosed usually in the sixth to seventh decade of life [39,40,41]. Diagnosing this type of pheochromocytoma is quite challenging, as we cannot rely on anatomical imaging modalities (MRI and CT). Accordingly, a recent study showed that 78% of pheochromocytomas with atypical anatomical imaging features (on CT or MRI) were totally or predominantly cystic [42]. In this situation, the most accurate way to assess these lesions is using a variety of functional imaging modalities like iodine metaiodobenzylguanidine (MIBG) scintigraphy, ^18^fluoro (F) deoxyglucose (FDG) positron emission tomography/computed tomography (PET/CT), ^18^FDG-PET/MRI, ^18^F-Fluoro-dihydroxyphenylalanine (^18^F-DOPA) PET/CT, and somatostatin receptor (SSTR) agonist-based PET/CT imaging [42,43]. It was demonstrated that even completely cystic pheochromocytomas captivate radiotracers like MIBG and FDG in the peripheral tumour rim or in residual tumour tissue [42]. Nevertheless, the current guidelines state that functional imaging should only be used if there is some biochemical evidence of catecholamine excess. On the other hand, there is no specific guidance for non-secretory pheochromocytomas, and their evaluation is dependent on an individualized approach [18,31]. Regarding our case, we decided not to perform functional imaging to avoid the unnecessary radiation exposure to a young asymptomatic patient with benign tumour features on MRI. 

The second obligatory test for evaluating all adrenal tumours is the 1 mg dexamethasone suppression test, which is conducted to rule out autonomous cortisol secretion. According to the newest guidelines for the management of adrenal incidentalomas, all patients with incidentally discovered adrenal masses should have this evaluation regardless of the clinical signs of hypercorticism [18]. In the largest series of benign adrenal cysts to date, autonomous cortisol secretion was suspected in 5% of patients [6], and it was not suspected in any patient in another study [3]. However, unlike the diagnostic challenges associated with cystic pheochromocytomas mentioned above, the biochemical confirmation of cortisol hypersecretion is usually straightforward. 

Testing for excess mineralocorticoids and androgens in a patient with any kind of adrenal tumour is not obligatory if there is no clinical suspicion [18]. Primary hyperaldosteronism in cases with unilateral adrenal masses is usually due to small (<2 cm) adrenal tumours, and, to our knowledge, there are no cases of benign cystic aldosterone-producing adenomas [44,45]. In the largest to-date cohort study of patients with benign adrenal cysts, primary hyperaldosteronism was suspected in 4% of patients, but it has not been confirmed with additional tests [6]. Accordingly, hypertension and hypokalaemia in the presence of a large cystic adrenal tumour should always raise the suspicion of aldosterone-secreting adrenocortical carcinoma (ACC) [46,47]. Similarly, hirsutism and/or virilization in the presence of a large cystic adrenal tumour may indicate the presence of a pure androgen-secreting adrenocortical tumour (PASAT) of which 50% are usually ACCs [48]. 

ACCs are rare malignant tumours with a reported annual incidence of 0.5–2 cases per million and a median age at presentation of 55 years [49]. The incidence of ACCs is still low even if the number of incidentally diagnosed cases is increasing (10–15% of cases) [49,50]. The typical appearance of ACC on CT/MRI is a large, heterogeneous solid tumour with irregular margins with the infiltration of adjacent anatomical structures, although intratumour haemorrhage may cause it to transform into a pseudocyst [51]. In 60% of cases, ACCs are hormonally functional tumours that usually secrete cortisol, but the secretion of oestrogen, androgens, and mineralocorticoids is well documented [49,50,51]. Clinicians typically consider PASAT or ACC when evaluating patients with hyperandrogenism and adrenal tumour, but they may overlook the possibility of congenital adrenal hyperplasia (CAH). 

CAH is a group of autosomal recessive disorders caused by the partial or complete deficiency of the enzymes involved in cortisol synthesis in the adrenal cortex [52]. Depending on the type of deficient enzyme, CAH can be a severe disease (classical CAH) manifested by cortisol and aldosterone deficiency with hyperandrogenism and virilization [52]. Patients with non-classical CAH (NC-CAH) have the residual activity of affected enzymes, and their disease is usually mild and presents mostly with hyperandrogenism [52]. However, either the complete or partial cortisol deficiency leads to a lack of negative feedback on the hypothalamic–pituitary–adrenal (HPA) axis, which increases ACTH production. ACTH stimulates unaffected enzymes involved in steroidogenesis in the adrenal cortex, leading to hyperandrogenism, but it is also a growth factor for adrenals, inducing their enlargement [53]. It is therefore common to discover bilateral adrenal hyperplasia on imaging in patients with CAH (especially the untreated ones). Nevertheless, it is unclear if ACTH also encourages tumour growth. A routine screening for adrenal tumours in patients with CAH is not recommended by the current guidelines [54], and a few studies investigated adrenal tumours in patients with CAH [53,55,56]. The overall prevalence of adrenal tumours in patients with CAH is estimated to be around 30% [52,53,55]. Additionally, the prevalence of CAH is higher in patients diagnosed with an adrenal tumour of any kind [57]. The most common adrenal tumours verified in CAH are myelolipomas [52,53,55,56,57], followed by adrenocortical adenomas [53,55,56,57]. However, there have been reported cases of pheochromocytomas [56,58] and ACCs [59]. More than half (60%) of patients with CAH and concomitant adrenal tumour had large tumours (more than 5 cm in diameter), and the majority were surgically treated [55,57]. Though larger tumours may deteriorate into pseudocysts, to our knowledge, there are no documented occurrences of adrenal cysts in patients with CAH. Considering that NC-CAH is often unrecognized, adrenalectomy performed in these patients may result in potentially fatal intra- or postoperative adrenal crisis. Furthermore, in an unrecognized CAH patient, adrenal insufficiency may manifest years after unilateral adrenalectomy [60]. Therefore, biochemical testing for adrenal androgens should be considered in all patients diagnosed with any kind of adrenal tumour. 

### 4.6. Question 6: Is There a Connection between Adrenal Cysts, Adrenal Insufficiency, and COVID-19?

Besides hormonal hyperfunction, all LATs (regardless of cystic transformation) can be associated with adrenal insufficiency, especially if they are primary or secondary malignant adrenal tumours or represent immune cell infiltrations, thrombosis, or haemorrhage seen in sepsis. Since our patient had recent COVID-19, we had to investigate the possibility that these two illnesses might be related. Although it is well established that SARS-CoV-2 has a notable affinity for the adrenal gland [61,62,63], to our knowledge, COVID-19 has not been linked to the development of adrenal cysts. The most prevalent pathology of the adrenal glands observed in patients with mild or severe COVID-19 was adrenal insufficiency caused by immune cell infiltrates and vascular damage, leading to thrombosis, infarctions, and haemorrhage [61,62,64,65,66]. Therefore, it would be reasonable to assume that adrenal pseudocysts might be visible on imaging; however, none of the published studies have discovered such presentation in COVID-19 patients [61,62,63,64,65,66,67]. A possible explanation could be the fulminant clinical course of severe COVID-19, which provides no time for structural disturbance to create an adrenal cystic lesion. Unfortunately, there are no long-term studies that analyse the imaging features and functionality of the adrenal glands in COVID-19 survivors who had an adrenal haemorrhage or thrombosis at the time of presentation. Our patient’s clinical presentation, normal adrenal function, and imaging characteristics of an adrenal cyst, along with the fact that this was her first-ever abdominal ultrasound, indicate that this non-functional endothelial adrenal cyst possibly existed before the patient’s COVID-19.

### 4.7. Question 7: To Operate or Not to Operate?

The decision for surgical or conservative treatment of a patient diagnosed with an adrenal cyst should be made according to the size and imaging characteristics of the lesion, the patient’s symptoms, and hormonal functionality. Approximately 50% of patients with adrenal cysts are being surgically treated, and most of them are usually asymptomatic [6,19]. According to the current guideline for the management of adrenal incidentalomas, asymptomatic, non-functional adrenal incidentalomas with benign imaging features and with (an arbitrary) diameter of ≤4 cm do not require surgery [18]. However, according to the same guidelines, larger adrenal tumours, with the same benign characteristics as mentioned above, require an individualized approach, mainly because of the concern both of the doctor and the patient, about the possibility of the increased incidence of malignancy in larger tumours (>4 cm) [11,12,18]. Nonetheless, we must remember that large cystic lesions, like the one in our case, could cause acute abdomen due to provoked or unprovoked bleeding into the cyst [68,69,70]. For adrenal cystic masses that have ambiguous imaging features, surgical treatment is usually preferred regardless of size and hormonal functionality [9]. 

A further issue is what kind of surgery should be performed. According to some case reports, adrenal cysts (with clear benign features on imaging) could be considered for aspiration. However, the reaccumulation of fluid is frequent and occurs in 30–50% of cases, even after sclerotherapy [4,5]. Laparoscopic adrenal cyst decortication or marsupialization could be other options [71]; however, these procedures rarely provide definitive healing. Percutaneous radiofrequency ablation (RFA) is a newly established procedure that could be effective and safe for the treatment of adrenal cysts [72]. When compared to percutaneous aspiration alone or in combination with sclerosing drugs, RFA appears to have a lower recurrence rate, making it a viable choice if a cyst returns following aspiration [72]. Surgical excision is still the usual approach for adrenal cyst management. It could be performed by simple cyst enucleation, but sometimes a partial or total adrenalectomy is needed. According to the guidelines for the management of adrenal incidentalomas, small adrenal tumours (≤2 cm) should be removed by using laparoscopic surgery, and this can be translated to the management of small adrenal cystic lesions, if they are considered for extirpation [18]. Since large adrenal tumours (>6 cm), including giant adrenal cysts, often adhere to surrounding tissue, laparoscopic resection in these cases could be associated with more complications, especially because of the insufficient operating space [73,74]. Additionally, laparoscopic resection of a suspected malignant cystic adrenal tumour could impose a risk for cyst puncture and the dissemination of cancer cells [75]. Nevertheless, some surgeons demonstrated the safety of laparoscopic surgery with the transperitoneal approach for large adrenal tumours up to 12–14 cm in diameter [76,77]. Regarding our case, we successfully extirpated an 8 cm large adrenal cyst using a transperitoneal laparoscopic approach, without injuring adjacent organs and managed to finish the surgery with an intact adrenal gland. Retroperitoneoscopy is another minimally invasive adrenal surgery technique performed usually on the apparently benign adrenal gland tumours that are up to 6 cm in diameter [78], although some centres practice this kind of surgery on adrenal masses of up to 8 cm [77]. The main advantages of the retroperitoneal laparoscopic approach are the direct access to the adrenal gland without the manipulation of other intraabdominal organs and the earlier recovery of the patients after the surgery [77,78]. However, this approach is demanding for a surgeon, as the operative field and visuality are limited [77]. 

In summary, although there is no specific guideline for the treatment of adrenal cysts yet, data from the literature, as well as the new proposal for the clinical management of patients with adrenal cysts by Calissendorff J et al., suggest that surgical resection should be performed when cysts are (a) symptomatic, (b) parasitic, (c) hormonally functional, and (d) have suspicious imaging features suggesting malignancy [4,9,79]. Although some surgeons advocate surgical treatment for clearly benign adrenal cysts that are larger than 5 cm [4,79], the new proposal for the clinical management of patients with adrenal cysts by Calissendorff J et al. suggests that the size of the cyst should not be a key feature for the decision to surgically treat these patients [9]. Adrenal cysts can continue to enlarge during the follow-up in one-third of the patients, while a decrease in the cyst size is rarely encountered [6,19]. If the decision for the follow-up has been made, the new imaging (CT or MRI) should be performed after 6–12 months or if needed (the development of mass effect symptoms or other new clinical signs or symptoms) [9]. However, in light of the lack of standardized protocols for the management of adrenal cysts, as well as an incomplete understanding of their pathogenesis and potential to grow and haemorrhage, treatment decisions should be individualized for every patient. 

## 5. Conclusions

The best course of treatment for adrenal cysts is still debatable because cases are infrequently reported, and there are several potential differential diagnoses. Before treating these lesions, it is crucial to rule out malignancy and hormonal functionality, but even the management of benign adrenal cysts is not standardized and is influenced by several variables, including the patient’s preferences. The size of the apparently benign adrenal cyst should not be the key feature for the decision to surgically treat these patients. However, we underline the word “apparently”, since even with modern imaging techniques, distinguishing between benign and malignant adrenal tumours is still challenging. Furthermore, the past case studies have shown us that large (>5 cm) adrenal cysts could provoke complications such as an acute abdomen and even may conceal an underlying malignancy. The morbidity and mortality related to these lesions have been greatly reduced as a result of the improvements in surgical methods and minimally invasive treatments. Therefore, we advise surgical treatment for young individuals with large, seemingly benign adrenal cysts but only after performing a thorough hormonal evaluation.

## Figures and Tables

**Table 1:** Results of hormonal analyses.

Hormonal Analyses	Patient’s Results	Ref. Value
Morning cortisol (nmol/L)	301	172–497
ACTH (pmol/L)	2.6	1.6–13
DHEAS (μg/dL)	185.9	65.1–368
Testosterone (nmol/L)	0.89	0.20–2.76
17OH-progesterone in luteal phase (ng/mL)	1.7	0.6–2.3
Aldosterone (ng/dL)	14.5	1.4–15.6
Plasma renin (μIU/mL)	37.8	2.8–39.9
Urinary metanephrine (μg/24 h)	112	<1000
Urinary normetanephrine (μg/24 h)	241	<600
Chromogranin A (ng/mL)	35.3	<100
Cortisol after overnight dexamethasone 1 mg (nmol/L)	14.9	<50

## Data Availability

Further inquiries can be directed to the corresponding author.
